# The benefits and harms of therapeutic exercise on physical and psychosocial outcomes in people with multimorbidity: Protocol for a systematic review

**DOI:** 10.1177/2235042X20920458

**Published:** 2020-05-12

**Authors:** Alessio Bricca, Lasse K Harris, Madalina Saracutu, Susan M Smith, Carsten B Juhl, Søren T Skou

**Affiliations:** 1 Research Unit for Musculoskeletal Function and Physiotherapy, Department of Sports Science and Clinical Biomechanics, University of Southern Denmark, Odense M, Denmark; 2 Department of Physiotherapy and Occupational Therapy, Næstved-Slagelse-Ringsted Hospitals, Region Zealand, Slagelse, Denmark; 3 HRB Centre for Primary Care Research, Department of General Practice, Royal College of Surgeons in Ireland (RCSI), Dublin, Ireland; 4 Department of Physiotherapy and Occupational Therapy, University Hospital of Copenhagen Herlev and Gentofte, Copenhagen, Denmark

**Keywords:** Multimorbidity, exercise, systematic review, meta-analysis, health-related quality of life, physical function, depression, anxiety

## Abstract

**Aim:**

The aim of this study is to investigate the benefits and harms of therapeutic exercise in people with multimorbidity defined as the combination of two or more of the following conditions: knee and hip osteoarthritis, hypertension, diabetes type 2, depression, heart failure, ischaemic heart disease and chronic obstructive pulmonary disease, by performing a systematic review of randomized controlled trials (RCTs).

**Methods:**

This study will be performed according to the recommendations from the Cochrane Collaboration and reported according to the Preferred Reporting Items for Systematic Reviews and Meta-Analyses (PRISMA). We will search for RCTs investigating the effect of therapeutic exercise in multimorbidity, as defined above, in MEDLINE, EMBASE, CENTRAL and CINAHL from 1990. Cochrane reviews on the effect of therapeutic exercise for each of the aforementioned conditions and references of the included studies will be checked for eligible studies and citation tracking will be performed in Web of Science. We will assess the risk of bias of the included studies using the Cochrane ‘Risk of Bias Tool’ 2.0 and the Grading of Recommendations Assessment, Development and Evaluation assessment for judging the overall quality of evidence. Meta-analyses will be performed, if possible, using a random-effects model as heterogeneity is expected due to differences in interventions and participant characteristics and outcome measures. Subgroup and meta-regression analyses will be performed to explore potential predictors of outcomes.

**Dissemination:**

The results of this systematic review will be published in a peer-review journal, presented at national and international conferences and made available to end users via infographics, podcasts, press releases and videos.

## Introduction

Knee and hip osteoarthritis (OA), hypertension, diabetes type 2 (T2DM), depression, heart failure, ischaemic heart disease and chronic obstructive pulmonary disease (COPD) are among the leading causes of global disability that affect hundreds of millions of people around the world.^1^ Recently, multimorbidity, defined by the World Health Organization as the coexistence of two or more chronic conditions, has been highlighted as *a priority for global health research*.^2^ Multimorbidity affects more than half of all people with a chronic condition^3^. For instance, two of the three people with OA have one or more other chronic conditions,^4^ with heart failure, ischaemic heart disease, hypertension, T2DM, COPD and depression being some of the most common.^5,6^ Compared to people living with only one chronic condition, people with multimorbidity are more likely to die prematurely, to be admitted to hospital and having an increased length of stay,^7,8^ in addition to having poorer physical function and quality of life, depression, intake of multiple drugs and increased healthcare utilization.^9–11^

Exercise is the cornerstone in the prevention of at least 35 chronic diseases and effective in the treatment of 26,^3^ including knee and hip OA,^12,13^ hypertension,^14^ T2DM,^15^ depression,^16^ heart failure,^17^ ischaemic heart disease^18^ and COPD.^19^ When exercise is used as treatment, it is often referred to as exercise therapy, which has been defined as ‘a regimen or plan of physical activities designed and prescribed for specific therapeutic goals with the purpose of restoring normal physical function or to reduce symptoms caused by diseases or injuries’.^20^

OA, hypertension, T2DM, depression, heart failure, ischaemic heart disease and COPD share a common risk factor (physical inactivity) and pathogenesis (systemic inflammation) which may trigger a cascade of reactions resulting in the development of a ‘vicious cycle’ of chronic diseases and poor outcomes.^21,22^ One of the key features of exercise is its anti-inflammatory effects at cellular, tissue and organ level.^21^ This highlights that the positive effects of therapeutic exercise may disrupt the ‘vicious cycle’ of chronic inflammation and improve physical and psychosocial health in people with multimorbidity. Furthermore, therapeutic exercise represents an intervention that is not necessarily condition-specific and such generic interventions are needed for patients with multimorbidity to reduce the treatment burden and disability.^23^

Several systematic reviews on the effect of therapeutic exercise have been performed focusing on individual conditions including OA, heart failure, ischaemic heart disease, hypertension, T2DM, COPD and depression. However, given that the impact of multimorbidity on the individual and society is larger than the impact of single chronic conditions alone,^8,24^ and that no systematic reviews on this topic has been identified, it is important to evaluate the effects of therapeutic exercise in people with multimorbidity.

Therefore, we aim to investigate the effects of therapeutic exercise in people with at least two of the aforementioned conditions on health-related quality of life, physical function and psychosocial outcomes by performing a systematic review of randomized controlled trials (RCTs). The systematic review will inform patients, researchers, clinicians and stakeholders on the efficacy of exercise therapy in people with multimorbidity and provide knowledge that will support the development of a therapeutic exercise intervention in people with multimorbidity, which will be tested in a later RCT in the MOBILIZE project (Text Box 1) (https://osf.io/qk6yg/).

Text Box 1 Contextual information for this systematic review.

**Figure 1.**
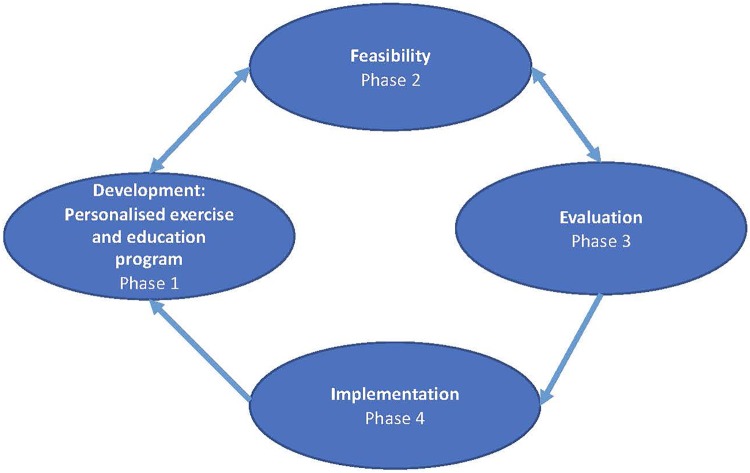
Overall framework for developing and evaluating complex interventions.

The aim of the MOBILIZE project is to empower patients with multimorbidity to take a more active role in their healthcare through a personalized exercise therapy and education programme aimed to reduce symptoms of the individual conditions, increase quality of life, physical function and prevent development of other chronic conditions. The MOBILIZE project comprises four phases and follows the MRC framework guidance for developing and evaluating complex interventions^28^ summarized in Figure 1. This systematic review is part of the development phase, together with three other studies; a scoping review mapping the behavioural change techniques used in patient-centred interventions for people with multimorbidity (https://osf.io/eszb7/), a systematic review to better understand the recruitment and retention process in RCTs including people with multimorbidity (https://osf.io/7bhcs/) and an observational cohort study from the Good Life with osteoArthritis in Denmark programme (GLA: D)^29^ to identify predictors of better health outcomes after a neuromuscular exercise and patient education programme in patients with osteoarthritis and one or more other conditions (https://osf.io/x8vja/).

## Methods

This protocol follows the Preferred Reporting Items for Systematic Reviews and Meta-Analyses Protocol (PRISMA-P) (Supplemental material 1) and a summary of the protocol (PROSPERO CRD42020150628), together with the statistical analysis plan (https://osf.io/zx9vw/) can be accessed via the Open Science Framework website.^25^ The study will be guided by the recommendations for performing systematic reviews in the Cochrane Handbook.^26^ The reporting will be performed according to the PRISMA guidelines.^27^

### Eligibility criteria for this systematic review

#### Study design

RCTs published in peer-reviewed journals or unpublished RCTs with available data.

#### Participants

We will include studies with at least 80% of participants reporting at least two of the following conditions: OA, heart failure, ischaemic heart disease, hypertension, T2DM, COPD and depression, as defined by the individual studies. We will also clearly report the numbers of all conditions for included studies.

#### Intervention

We will include therapeutic exercise interventions with or without additional pharmacotherapy or other adjuvant interventions (e.g. weight loss) as long as these co-interventions are delivered to both the intervention group and the comparator group. We will include interventions targeting two of the conditions of interest and interventions targeting only one of the conditions of interest as long as the participants meet the inclusion criteria for this systematic review (i.e. at least 80% of participants with multimorbidity).

We will exclude interventions without a structured therapeutic exercise programme delivered (e.g. providing a pedometer or booklet to the participants without a specific plan for therapeutic exercise).

#### Comparator

Usual/standard care (at the time the trial was conducted) and comparator groups non-exposed (e.g. wait-and-see and placebo treatments).

### Outcomes

#### Main outcomes

- Self-reported and objectively measured physical function.
- Health-related quality of life.
- Psychosocial outcomes: anxiety and depression.
- Adverse events as reported in included trials and grouped as serious or non-serious according to the FDA definition.^30^

#### Timing and effect measures

Data will be extracted at all time points available with clear recording of the pre-specified end point for each study. For this systematic review, end treatment end points (immediately after the intervention) and as close to 12 months as possible will be the end points included in the primary analyses.

### Information sources

We will obtain information from three sources:

1. Searching MEDLINE via PubMed, EMBASE via Ovid, CINAHL (including pre-CINAHL) via EBSCO and the CENTRAL with no restriction on language. We will only include RCTs published from 1990 since the reporting as well as the treatment of multimorbidity has changed substantially within the last years.
2. Screening the reference lists of the latest Cochrane reviews investigating the effect of therapeutic exercise on the following conditions: osteoarthritis, hypertension, diabetes type 2, depression, heart failure, ischemic heart disease, chronic obstructive pulmonary disease.
3. Screening the reference lists of included RCTs.
4. Screening for completed trials in the World Health Organization’s International Clinical Trials Registry Platform (ICTRP; http://apps.who.int/trialsearch/), which comprises the 16 primary registries of the WHO registry network and ClinicalTrials.gov.
5. Searching Web of Science for studies citing the RCTs included in this systematic review (citations tracking).

### Search strategy

The search strategy was developed for MEDLINE (Supplemental material 2) and was customized for EMBASE, CINAHL and CENTRAL. All terms were searched, if possible, both as keywords (Mesh) and as text words in title and abstracts (TIAB). To identify RCTs we used the Cochrane-sensitive search strategy for identifying RCTs.^26^

### Data management and selection process

The results of the literature search will be uploaded to EndNote X9.3.1.^31^ Two reviewers will independently screen titles and abstracts, and all studies deemed eligible by at least one of the reviewers will be checked independently in full text by two reviewers. Disagreement between the reviewers about inclusion of individual studies will be discussed until consensus has been reached. If disagreement persists, a third reviewer will be contacted to resolve the disagreement. We will check whether multiple reports from the same study are published by juxtaposing author names, treatment comparisons, sample sizes and outcomes. If multiple reports of the same studies provide different study characteristics (e.g. number of participants and presence of one or more chronic conditions), we will use the primary publication. We will record the reasons for excluding full-text RCTs. Neither of the review authors will be blind to the journal titles, study authors or research department.

### Data collection process

We will use the Cochrane Collaboration data collection form for intervention reviews: RCTs only^32^ available here: https://osf.io/tpqjb/. We will extract the following data for continuous outcomes (number of participants, mean and standard deviation, standard error or 95% confidence interval) and for categorical outcomes (cases and total number of participants) depending upon availability of the data:

- Trial characteristics: design of trial (e.g. factorial), location of the trial (in case of multicentre studies, primary investigator affiliation will apply), number of patients allocated (to the exercise and comparator groups respectively), number of patients in the intention to treat and per-protocol analysis (in the intervention and comparator groups, respectively).
- Participant characteristics: age, % female, body mass index, ethnicity, socioeconomic status (studies will be labelled as low SES when the majority of the participants are described as having low education levels, low income, being unemployed or sample otherwise labelled as ‘low SES’), blood biomarkers (hs-C-reactive protein, tumour necrosis factor and interleukin 6, HbA1c, triglycerides, high-density lipoprotein and low-density lipoprotein), baseline severity and diagnosis of the conditions, and number, type and frequency of other conditions.
- Intervention and comparator characteristics: Components of intervention (e.g. therapeutic exercise + diet or only exercise), type of intervention/comparator interventions (aerobic, neuromuscular, strengthening or a combination of those), frequency of the sessions (times per week), intensity of the session (% of maximum pulse, or % of 1 Repetition Maximum), volume of the sessions, mode of delivery (one-to-one, group, or a combination of these), setting (home-based, clinic-based, or a combination of these), duration of the interventions (in weeks), supervision (yes, no or a combination), tailoring (intervention developed according to guidelines and individual patients’ needs), adherence to intervention (number of sessions attended out of the total number of planned sessions), use of financial incentives for intervention adherence or follow-up assessment attendance, modifications of the intervention during the study.
- Outcome characteristics: time points assessed and the magnitude of objectively and subjectively measured changes (e.g. change in health-related quality of life, number of adverse events in the intervention and comparator groups). Additionally, we decided how to prioritize outcome measures for extracting data from the included RCTs. We will prioritize data extraction of outcome measures important for the participants and generic over disease-specific measures. For objectively measured physical function, we will prioritize: (1) the 6-minute walking test, (2) Incremental Shuttle Walking Test, and (3) any other outcome measure related to daily function (e.g. chair stand test). For self-reported physical function, we will prioritize: (1) the 36-item Short-Form Health Survey (SF-36) Physical Function subscale, (2) the SF-36 Role Function subscale, and (3) any other self-reported measure of physical function. For health-related quality-of-life outcomes, we will prioritize: (1) the EuroQol (EQ-5D) questionnaire, (2) any other health-related quality-of-life questionnaires, (3) disease-specific health-related quality-of-life questionnaires (e.g. The Minnesota living with heart failure questionnaire). For depression, we will prioritize: (1) The Beck Depression Inventory, (2) any other depression questionnaire (e.g. the Hospital Anxiety and Depression Scale (HADS depression). For anxiety, we will prioritize: (1) State Trait Anxiety Inventory questionnaire and (2) any other anxiety questionnaire (e.g. HADS anxiety).

Authors will be contacted if the data cannot be extracted from the published manuscripts.

### Risk-of-bias assessment

Two reviewers (Alessio Bricca and Lasse K Harris) will independently assess the methodological quality of the included studies using the Cochrane ‘Risk of Bias Tool 2.0’.^26^ Bias will be assessed in five distinct domains: Bias arising from the randomization process or lack of allocation concealment, bias due to deviations from intended interventions, bias due to missing outcome data, bias in measurement of the outcome or delivery of the intervention (blinding) and bias in selection of the reported result. Within each domain, the two reviewers will answer one or more signalling questions (e.g. Was the allocation sequence random? and Were participants aware of their assigned intervention during the trial?), which will lead to judgements of ‘low risk of bias’, ‘some concerns’ or ‘high risk of bias’. The judgements within each domain lead to an overall risk-of-bias judgement for the result being assessed. The overall quality of evidence for the estimates will be evaluated using the Grading of Recommendations Assessment, Development and Evaluation (GRADE) approach.^33^ The GRADE is a systematic approach to rate the quality of evidence across studies for specific outcomes. It is based on five domains that involve the methodological flaws of the studies (i.e., risk of bias), the heterogeneity of results across studies (i.e., inconsistency), the generalizability of the findings to the target population (i.e. indirectness), the precision of the estimates and the risk of publication bias.

### Data synthesis

Pre-specified statistical analyses are available at the Open Science Framework website at this link (https://osf.io/zx9vw/). Overall, we will perform one meta-analysis for each outcome of interest. For physical function, we will perform a stratified meta-analysis on objectively and self-reported outcomes. We will use a random-effects model as heterogeneity is expected due to differences in interventions, outcome measures and so on. Heterogeneity will be examined as between-study variance and calculated as the *I* ^2^ statistic measuring the proportion of variation in the combined estimates due to between-study variance. An I-squared value of 0% indicates that no inconsistency between the results of individual trials, and an *I* ^2^ value of 100% indicates maximal inconsistency. Standardized mean differences (SMDs) with 95% CIs will be calculated for outcome measures of continuous data and adjusted to Hedges *g*. The magnitude of the effect size of the pooled SMD will be interpreted as 0.2, representing a small effect, 0.5 a moderate effect and 0.8 a large effect.^34^ For outcome measures where a meta-analysis is not possible, a narrative data synthesis of the results from individual studies will be performed in line with the guidance from the Cochrane handbook.^26^

### Analysis of subgroups or subsets

Subgroup analyses will be performed to explore whether the effect of exercise varies in people with different combinations of chronic conditions. Furthermore, we will investigate the impact of risk of bias on the estimates for the outcomes of interest by classifying studies at ‘low risk of bias’, ‘some concerns’ or ‘high risk of bias’ according to the Cochrane Risk of Bias Tool 2.0. Meta-regression analyses will also be performed to identify factors (covariates) which predict better health outcome. Relevant study-level covariates are defined as ones able to decrease inconsistency measured as the *I* ^2^ statistic (and thus the between-study variance *τ* ^2^).^26^ Based on a systematic screening of the latest Cochrane systematic reviews investigating the effect of therapeutic exercise on the individual conditions of interest (https://osf.io/zx9vw/) and input from members of the study team, we identified factors that may predict better outcomes in therapeutic exercise trials. These factors are listed in the Data collection process paragraph.

## Conclusions and implications

This protocol details the plan for a systematic review investigating the effect of therapeutic exercise in people with multimorbidity. Given that the impact of multimorbidity on the individual and society is larger than the impact of a single chronic condition alone^8,24^ and the fact that a systematic summary for the effect of exercise in this population has not been performed, this systematic review will serve to inform end users and researchers on the efficacy of exercise in people with multimorbidity. Additionally, it will be a cornerstone in the development of an exercise intervention that will be tested in the MOBILIZE project.

## Supplemental material

Supplemental Material, PRISMA-P - The benefits and harms of therapeutic exercise on physical and psychosocial outcomes in people with multimorbidity: Protocol for a systematic review Click here for additional data file. Supplemental Material, PRISMA-P for The benefits and harms of therapeutic exercise on physical and psychosocial outcomes in people with multimorbidity: Protocol for a systematic review by Alessio Bricca, Lasse K Harris, Madalina Saracutu, Susan M Smith, Carsten B Juhl and Søren T Skou in Journal of Comorbidity

Supplemental Material, PubMed_search_strategy - The benefits and harms of therapeutic exercise on physical and psychosocial outcomes in people with multimorbidity: Protocol for a systematic review Click here for additional data file. Supplemental Material, PubMed_search_strategy for The benefits and harms of therapeutic exercise on physical and psychosocial outcomes in people with multimorbidity: Protocol for a systematic review by Alessio Bricca, Lasse K Harris, Madalina Saracutu, Susan M Smith, Carsten B Juhl and Søren T Skou in Journal of Comorbidity
