# Supplementary material for: The benefits and harms of therapeutic exercise on physical and psychosocial outcomes in people with multimorbidity: Protocol for a systematic review
Source: J Comorb. 2020 May 12;10:2235042X20920458. doi: 10.1177/2235042X20920458 (PMC7218324; doi:10.1177/2235042X20920458)
Supplement: Supplemental Material, PubMed_search_strategy - The benefits and harms of therapeutic exercise on physical and psychosocial outcomes in people with multimorbidity: Protocol for a systematic review [file PubMed_search_strategy.pdf]

Supplementary 2

PubMed search strategy.

**Recent queries in pubmed**		
**Search**	Query	Items found
**#17**	Search (#14 AND #15 AND #16)	13484
**#16**	Search (((randomized controlled trial[pt] OR controlled clinical trial[pt] OR randomized[tiab] OR placebo[tiab] OR drug therapy[sh] OR randomly[tiab] OR trial[tiab] OR groups[tiab] NOT (animals[mh] NOT humans[mh]))))	3991357
**#15**	Search ((((Exercise[MeSH] OR Exercis*[tiab] OR Walking[MeSH] OR Walk*[tiab] OR Running[MeSH] OR Run*[tiab] OR Muscle Contraction[MeSH] OR Strengthening[tiab] OR Cycling[tiab] OR Weight lifting[MeSH] OR Weight lifting[tiab] OR Jogging[MeSH] OR Jogging[tiab] OR Swimming[MeSH] OR Swimming[tiab] OR Pool therapy[tiab] OR Aquatic exercise[tiab] OR Hydrotherapy[MeSH] OR Hydrotherapy[tiab] OR Gymnastic[MeSH] OR Gymnastic*[tiab] OR physical activity[mesh] OR physical activity[tiab] OR Resistance Training[mesh] OR Resistance Training[tiab] OR weight-bearing[mesh] OR weight-bearing[tiab] OR endurance Training[tiab] OR muscle strength[mesh] OR muscle strength[tiab] OR exercise therapy[mesh] OR exercise therapy[tiab] OR Physical Therapy Modalities[mesh] OR Physiotherapy[tiab] OR Physical Therapy[tiab]))))))	1090668
**#14**	Search (#8 OR #9 OR #10 #11 OR #12 OR #13)	285810
**#13**	Search (#7 AND (#2 OR #3 OR #4 OR #5 OR #6 OR #1))	207921
**#12**	Search (#6 AND (#2 OR #3 OR #4 OR #5 OR #1 OR #7))	14585
**#11**	Search (#5 AND (#2 OR #3 OR #4 OR #1 OR #6 OR #7))	212955
**#10**	Search (#4 AND (#2 OR #3 OR #1 OR #5 OR #6 OR #7))	77790
**#9**	Search ((#3 AND (#2 OR #1 OR #4 OR #5 OR #6 OR #7)))	124116
**#8**	Search (#1 AND (#2 OR #3 OR #4 OR #5 OR #6 OR #7))	5133
**#7**	Search (((Myocardial Ischemia[mesh]) OR (Myocardial Ischemia[tiab]) OR (Coronary Artery Disease[mesh]) OR (Coronary Artery Disease[tiab]) OR (Coronary Disease[mesh]) OR (Coronary Disease[tiab]) OR (Myocardial Infarction[mesh]) OR (Myocardial Infarction[tiab]) OR (Angina Pectoris[mesh]) OR (Angina Pectoris[tiab]) OR (Heart Failure[mesh]) OR (Heart Failure[tiab]) OR (HFNEF[Title/Abstract] OR HFPEF[Title/Abstract] OR HFREF[Title/Abstract] OR "HF NEF"[Title/Abstract] OR "HF PEF"[Title/Abstract] OR "HF REF"[Title/Abstract]) OR (Heart Diseases[mesh]) OR (Heart Diseases[tiab]) OR (Coronary Artery Bypass[mesh]) OR (Coronary Artery Bypass[tiab]))))	1222567
**#6**	Search (((Pulmonary Disease, Chronic Obstructive[mesh]) OR (COPD[mesh]) OR (COPD[tiab]) OR (Pulmonary Emphysema[mesh]) OR (Pulmonary Emphysema[tiab]) OR (COAD[mesh]) OR (COAD[tiab]) OR (Bronchitis, Chronic[mesh]) OR (Chronic Bronchitis[tiab]) OR (Chronic Obstructive Lung Disease[tiab])))	78618
**#5**	Search (((Hypertension[MeSH]) OR (Hypertension[tiab]) OR (Hypertens*[tiab]) OR (High Blood Pressure[tiab]) OR (Blood Pressure[mesh])))	678932
**#4**	Search (((depression[mesh]) OR (depression[tiab]) OR (Dysthymic Disorder[mesh]) OR (Dysthymic Disorder[tiab]) OR (depress*[tiab]) OR (dysthymi*[tiab]) OR (affect disorder*[tiab]) OR (affective symptom*[tiab])))	482492
**#3**	Search (((diabetes mellitus[mesh]) OR (diabetes mellitus[tiab]) OR (Diabetes Mellitus, Type 2[mesh]) OR (NIDDM[tiab]) OR (impaired glucose toleranc*[tiab]) OR (Glucose Intolerance[MeSH]) OR (Blood Glucose[MeSH])))	566405
**#2**	Search (((((((((((((((((((((((((co-existing health problem*[tiab]) OR coexisting illness*[tiab]) OR co-existing illness*[tiab]) OR coexisting patholog*[tiab]) OR co-existing patholog*[tiab]) OR comorbid condition*[tiab]) OR co-morbid condition*[tiab]) OR comorbid diagnos*[tiab]) OR co-morbid diagnos*[tiab]) OR comorbid disease*[tiab]) OR co-morbid disease*[tiab]) OR comorbid illness*[tiab]) OR concurrent disease*[tiab]) OR co-occurring condition*[tiab]) OR cooccurring condition*[tiab]) OR "multiple conditions"[tiab]) OR "multiple disease"[tiab]) OR "multiple diseases"[tiab]) OR Comorbidity[Mesh]) OR Comorbidity[tiab]) OR "comorbidities"[tiab]) OR multimorbidity[tiab]) OR multimorbidity[mesh]) OR multi-morbidity[tiab]) OR multi-morbidities[tiab]))	204394
**#1**	Search ((((((((Osteoarthritis[mesh]) OR (Osteoarthritis[tiab]) OR (Osteoarthrit*[tiab]) OR (Osteoarthros*[tiab]))))	85990
